# Factors Influencing Acceptance of Microalgae‐Based Iron Supplements in Romanian Adults With Self‐Reported Iron‐Deficiency Anemia: A Cross‐Sectional Study

**DOI:** 10.1155/anem/3201387

**Published:** 2026-09-08

**Authors:** Alexandra Lacurezeanu, Dan Cristian Vodnar

**Affiliations:** ^1^ Faculty of Food Science and Technology, University of Agricultural Sciences and Veterinary Medicine, Cluj-Napoca, Romania, usamvcluj.ro; ^2^ Institute of Life Sciences, University of Agricultural Sciences and Veterinary Medicine, Cluj-Napoca, Romania, usamvcluj.ro

**Keywords:** complementary medicine, Health Belief Model, iron-deficiency anemia, microalgae, patient acceptance, Romania

## Abstract

Microalgae‐derived iron preparations are entering clinical discourse as a potentially better‐tolerated alternative to conventional ferrous salt supplementation, which fails a substantial proportion of the approximately 571 million women globally affected by iron‐deficiency anemia (IDA) due to gastrointestinal adverse effects. Yet translational readiness requires more than biological efficacy data it requires knowing whether patients would actually accept such preparations under realistic informational conditions. No study has addressed this question in a defined IDA population. We conducted a cross‐sectional online survey among 71 Romanian adults with physician‐diagnosed IDA and prior oral iron experience, measuring informed acceptance assessed only after participants received standardized clinical information, including current evidence limitations, using a validated 5‐item composite score (ACC_score; *α* = 0.801). Health Belief Model (HBM) constructs, complementary and alternative medicine (CAM) attitudes, motivators, adverse‐effect burden, and physician trust were examined as predictors via Spearman correlations and ordinary least squares regression. Acceptance was high overall (ACC_score = 20.68, SD = 3.25) though concentrated near the scale ceiling (IQR = 19–23), limiting variability in the outcome and likely attenuating observed associations. The multivariate model explained 63.8% of variance (*p* < 0.001); self‐efficacy showed the strongest standardized association in the primary OLS model (*β* = 0.384), followed by perceived barriers (*β* = −0.275), CAM attitudes (*β* = 0.248), and perceived susceptibility (*β* = 0.229) though self‐efficacy did not reach statistical significance in a binary logistic sensitivity analysis (OR = 4.01, *p* = 0.058), most likely reflecting information loss from outcome dichotomization. Critically, among individual barrier items, only the lack of long‐term evidence reached statistical significance a specific, addressable target for patient communication. Notably, safety concerns about current iron formulations were positively, not negatively, associated with acceptance, consistent with an activated patient profile in which dissatisfaction with tolerability motivates alternative‐seeking. The sample was highly selected (95.8% female and 91.5% university educated), and the findings should be interpreted as hypothesis‐generating and require confirmation in larger, clinically verified, and more representative IDA populations.

## 1. Introduction

Iron‐deficiency anemia (IDA) remains among the most prevalent and burdensome nutritional disorders globally, disproportionately affecting women of reproductive age and representing one of the leading contributors to disability‐adjusted life years across both high‐ and low‐income settings [1–3]. Global Burden of Disease estimates indicate that anemia affected hundreds of millions of women of reproductive age in 2019, with substantial regional variation and a persistently higher burden in South Asia and sub‐Saharan Africa [1, 4]. Romania is not exempt from this burden: population‐level estimates indicate that approximately 22.6% of nonpregnant women aged 15–49 years were affected by anemia, a figure that has remained relatively stable since 2000 [5]. Nationally representative IDA‐specific prevalence data for Romanian adults remain limited, as population‐level surveillance has primarily focused on women of reproductive age and pediatric populations. IDA is defined by the World Health Organization as hemoglobin levels below 12 g/dL in nonpregnant women and 13 g/dL in men, combined with evidence of iron depletion [6]. Iron deficiency is the most common nutritional cause of anemia, driven by inadequate dietary iron intake and bioavailability, excessive menstrual blood loss, recurrent pregnancy, and impaired gastrointestinal absorption, with inflammation and infection further compounding functional iron deficiency in many settings [7–9]. Beyond hematological consequences, IDA is associated with fatigue, reduced physical work capacity, impaired cognitive performance, diminished quality of life, and adverse reproductive outcomes, including increased risk of preterm birth, low birth weight, and perinatal mortality [1, 2, 10]. Despite the availability of effective treatments, the persistence of IDA is driven by multifactorial barriers, including poor adherence to oral iron due to gastrointestinal tolerability, health system constraints, social determinants such as poverty and food insecurity, and gaps in screening and provider knowledge, rendering progress toward global anemia reduction targets critically insufficient [8, 11].

Oral iron supplementation with ferrous salt preparations remains the first‐line treatment for IDA due to their low cost and wide availability; however, gastrointestinal tolerability is a critical, well‐documented limitation [12, 13]. A systematic review and meta‐analysis of 43 randomized controlled trials (*n* = 6831) demonstrated that ferrous sulfate significantly increased the risk of gastrointestinal adverse effects compared with placebo (OR = 2.32, 95% CI [1.74–3.08], *p* < 0.0001), including nausea, constipation, abdominal cramping, diarrhea, bloating, and metallic taste [14]. These adverse effects are not limited to standard dosing regimens: even low‐dose prophylactic iron supplementation formulations have been associated with gastrointestinal complaints in some populations although the clinical significance varies by formulation and dose [15]. Across clinical settings, gastrointestinal intolerance frequently prompts dose reduction, treatment switching, or discontinuation, representing a recognized and modifiable driver of suboptimal adherence to iron therapy [12, 14]. The clinical and economic consequences are substantial: persistent iron deficiency sustains productivity losses estimated at 12 US dollars of potential return for every dollar invested in anemia reduction [16].

Microalgae, especially *Arthrospira platensis* (Spirulina) and *Chlorella vulgaris*, have gained increasing research attention as naturally sourced, bioavailable providers of iron, embedded within an organic nutritional matrix that includes vitamins B12, C, and folate [17]. A recent PRISMA‐compliant systematic review of 32 in vivo studies identified consistent improvements in hematological parameters following Spirulina and Chlorella supplementation [17]. A randomized, double‐blinded, placebo‐controlled trial in 80 adults with ulcerative colitis found that 8 weeks of Spirulina supplementation (1 g/day) significantly increased serum iron levels (*p* = 0.04), with no adverse reactions [18]. Despite this early preclinical and small‐scale clinical evidence, patient acceptance of microalgae‐derived iron supplements has not been systematically studied in a clinically defined IDA population, and data from Eastern European healthcare contexts are absent. Understanding the belief‐based determinants of acceptance is a necessary precursor to any future clinical translation of these preparations [17, 19].

Health Belief Model (HBM) offers a useful framework for examining belief‐based determinants of health‐related decision‐making, incorporating perceived susceptibility, perceived severity, perceived benefits, perceived barriers, self‐efficacy, and cues to action [19, 20]. In the context of iron supplementation specifically, HBM constructs have been applied across diverse populations: Lotfipur et al. (2024) identified self‐efficacy and perceived severity as significant independent predictors of iron supplement consumption behavior among children; Silitonga et al. (2023) further confirmed, in a systematic review, that perceived barriers, self‐efficacy, and cues to action were the primary determinants of iron supplement‐taking behavior among adolescent girls; and Athayalillah et al. (2024) demonstrated that HBM‐derived belief constructs, alongside nutritional knowledge, shaped supplement consumption decisions among young women preparing for marriage [21–23].

The HBM has been applied in Romanian and Eastern European contexts to examine preventive health behaviors, including during the COVID‐19 pandemic [24], providing precedent for its use in this population, though direct validation in nutritional supplementation contexts in Eastern Europe remains limited. The specific objectives of the present study were (1) to characterize informed acceptance following the provision of standardized product information, (2) to identify HBM‐consistent and related factors independently associated with acceptance, and (3) to determine whether acceptance scores varied across selected sociodemographic and clinical subgroups. To the best of our knowledge, no prior research has explored this issue within an adult IDA population in Romania.

## 2. Methods

### 2.1. Study Design and Participants

This study employed a cross‐sectional, observational design using a self‐administered online questionnaire, conducted in Romania in 2026 and reported in accordance with the Strengthening the Reporting of Observational Studies in Epidemiology (STROBE) guidelines [25, 26]. A completed STROBE checklist is provided as supporting information. Cross‐sectional online surveys are increasingly used to study populations with chronic diseases because they are cost‐effective and can reach dispersed patient groups. However, such surveys have well‐known methodological limitations, including participation bias, nonresponse bias, and the potential over‐representation of individuals who are more engaged with healthcare or digital health settings. All six HBM constructs were incorporated as predictors to prevent selective exclusion.

Participants were eligible if they (1) were 18 years or older; (2) reported having received a physician‐confirmed diagnosis of IDA (self‐reported clinician diagnosis; clinical records were not verified by the research team); and (3) had current or prior experience with oral iron supplementation. Individuals with non‐IDA causes of anemia, those under 18, and those who did not provide informed consent were excluded. One participant was excluded due to age, resulting in a final analytic sample of *n* = 71. Male participants were not excluded a priori, as IDA occurs across sexes and exclusion would have introduced an additional layer of selection bias beyond that already inherent in convenience sampling; however, with only three male participants (4.2%), the sample is effectively female dominant and findings should not be assumed to apply to men, consistent with the SAGER limitations noted in Section 5. No data were missing for any participant on variables included in the analyses (complete case rate = 100%). The complete participant flow, including eligibility screening and exclusions, is presented in Supporting Figure S1 [27, 28].

Recruitment was conducted via convenience sampling through patient communities, online groups, and healthcare professional referral channels, primarily operating online in Romania. Convenience sampling, while pragmatically necessary in exploratory research, systematically limits external validity and may bias sample composition by age, sex, education, disease severity, and digital access; findings should, therefore, be interpreted with explicit acknowledgment of these constraints [27–29]. Participation was voluntary, anonymous, and uncompensated. It should be noted that the survey platform did not record the total number of individuals who encountered the survey link; consequently, a response rate cannot be calculated for this study, which is a recognized limitation that precludes assessment of nonresponse bias and should be considered when interpreting the representativeness of the findings. All participants provided electronic informed consent prior to data collection. The study received approval from the Bioethics Committee of the University of Agricultural Sciences and Veterinary Medicine Cluj‐Napoca (Decision No. 535/24.10.2025; application no. 522/20.10.2025), in compliance with the Declaration of Helsinki and Regulation (EU) 2016/679 (GDPR). Participant anonymity was maintained through pseudonymized codes (P001–P071). The IDA status was based on participants’ self‐reports of prior clinician diagnoses and was not independently verified against medical records; diagnostic misclassification is, therefore, possible, a recognized limitation of online cross‐sectional studies that rely on self‐reported clinical diagnoses [27, 30].

### 2.2. Questionnaire and Measures

The questionnaire was administered in Romanian in two consecutive parts. In the initial section, participants answered questions about their clinical history, HBM constructs, and other covariates. Before encountering any acceptability questions, all participants received a standardized factual description of microalgae‐based iron supplements, covering biological sources, iron levels, bioavailability mechanisms, nutritional cofactors (vitamins B12, C, and folate), a summary of current clinical evidence, including its limitations, such as limited long‐term data, and safety considerations.

Only after reviewing this standardized information did participants proceed to the acceptability questions. This sequence is crucial: the ACC_score assesses informed acceptance intention, the cognitive evaluation of a well‐described option within a consistent informational context, not a response to an abstract idea. Framing acceptance outcomes in relation to a specific, well‐defined intervention is consistent with best practice in HBM‐based questionnaire design, where items should be explicitly tied to the health intervention under study to maximize predictive validity [30, 31].

The questionnaire was based on established HBM operationalizations of health behavior intention and treatment adherence [32]. All six HBM constructs were implemented as recommended in the literature to avoid selective omission [30, 33]: perceived severity (Items 49 + 26; HBM_sev range 2–10); perceived susceptibility (Item 50; HBM_sus range 1–5—single item, due to the absence of a validated multi‐item Romanian IDA‐specific susceptibility scale); perceived benefits (Item 51; HBM_ben range 1–5—single item, for the same reason); self‐efficacy (Items 52–53; HBM_se range 2–10; *α* = 0.834); and perceived barriers (Items 44–48, range 4–20 after excluding Item 46; see below). The use of single‐item measures for HBM_sus and HBM_ben is a recognized limitation: multi‐item subscales are preferred to enhance reliability and construct validity and single‐item indicators may attenuate observed associations due to measurement error [34–36]. Additional constructs included motivators (Items 37–43; Motiv_score range 7–35), trust in physician (I58; “I trust my doctor’s recommendations regarding supplements;” 1 = *strongly disagree* and 5 = *strongly agree*), and trust in science (I59; “I trust scientific research as a basis for health decisions;” 1 = *strongly disagree* and 5 = *strongly agree*). The burden of gastrointestinal adverse effects (Items 12–17) was assessed using a six‐item scale (AE score range 0–36; *α* = 0.806), and attitudes toward complementary and alternative medicine (Items 27–31) were evaluated on 5‐point Likert scales (CAM_score range 5–25; *α* = 0.838).

Two methodological issues regarding the barriers scale should be acknowledged. First, Item 46 (cost as a barrier) was mistakenly recorded as a binary variable due to a formatting error on the survey platform. As a result, it was excluded from the barriers composite (BAR_comp) and analyzed separately with the Mann–Whitney *U* test. Sensitivity analysis that included I46 as a binary covariate in the primary regression confirmed that its exclusion did not significantly alter the main results (*B* = −0.214, SE = 0.545, *p* = 0.697; change in *R* ^2^ < 0.001). Second, the BAR_comp (I44, I45, I47, I48; range 4–20) showed low internal consistency (*α* = 0.601, below the acceptable threshold of 0.70 [37]), suggesting conceptual heterogeneity among barrier types. Psychometric research indicates that perceived barriers can be multidimensional and that low internal consistency may suggest analyzing individual barrier items rather than treating them as a single, unidimensional scale [30, 34, 38]. M1, which decomposes BAR_comp into individual barrier items, was prespecified alongside M0 as a prespecified exploratory sensitivity model: M0 ensures complete HBM theoretical coverage using the composite; M1 provides disaggregated barrier estimates that address the reliability limitation (*α* = 0.601). M1 findings should, therefore, be interpreted as exploratory and hypothesis‐generating. The questionnaire was reviewed for face validity by two subject‐matter experts prior to deployment; no full pilot study was conducted, which likely delayed identification of the Item 46 formatting error. To address concerns about the integrity of the remaining items, all variables other than Item 46 were inspected for implausible values, out‐of‐range responses, and distributional anomalies prior to analysis; no additional irregularities were identified, and all remaining items were confirmed to have been recorded on their intended Likert scales with complete data.

The primary outcome, informed acceptance (ACC_score), was measured using five items (I32–I36) administered after the standardized information text. These items assessed willingness to try, the likelihood of acceptance if recommended by a healthcare professional, the probability of trying within 6 months, willingness to recommend to others, and willingness to participate in a clinical trial. Each item was rated on a 5‐point ordinal scale (ranging from 5 to 25), with a Cronbach’s *α* of 0.801. Corrected item‐total correlations ranged from 0.46 (I36, clinical trial participation) to 0.71 (I32, willingness to try), showing all items contributed meaningfully to the overall score. Following guidance on HBM‐based outcome measurement, ACC_score reflects informed acceptance intention rather than actual product uptake, acknowledging the common intention–behavior gap in adherence research [31, 33].

### 2.3. Statistical Analysis

All statistical analyses and figures were performed exclusively in JASP (Version 18; JASP Team, 2024). Normality of all continuous variables was assessed using the Shapiro–Wilk test [39]. As all variables showed significant departures from normality (all *p* < 0.05), Spearman rank correlations were used for all bivariate analyses. This choice is consistent with methodological guidance indicating that Spearman’s *ρ* is preferred over Pearson’s *r* when variables are nonnormally distributed, ordinal, or when the hypothesized relationship is monotonic rather than strictly linear, as it relies on ranks rather than raw values and is less sensitive to distributional violations [38]. For the multivariate analyses, ordinary least squares (OLS) regression was employed despite the nonnormality of the original variable distributions. OLS regression requires normally distributed residuals rather than normally distributed predictors or outcomes; when residual diagnostics confirm this assumption, OLS yields unbiased and consistent estimates of mean relationships even when the underlying variables are nonnormal [40]. This approach is appropriate when the dependent variable is treated as approximately continuous as is the case for a five‐item composite scale with a sufficiently wide range and when residual diagnostics confirm acceptable linearity, homoscedasticity, and normality of errors [41, 42]. Residual normality was confirmed for both models via Shapiro–Wilk tests on the model residuals (see Section 3.4), thereby satisfying the core OLS assumption. To ensure full transparency, the following OLS assumptions were verified prior to interpretation: (1) residual normality was assessed via the Shapiro–Wilk test on model residuals and confirmed for M0 (SW *W* = 0.972, *p* = 0.110); (2) homoscedasticity and linearity were evaluated via the residuals‐versus‐predicted‐values plot (Supporting Figure S3), which showed no systematic pattern; and (3) multicollinearity was assessed via VIF for all predictors (all VIF < 3; see Section 3.4). These diagnostics collectively confirm that the fundamental assumptions of OLS regression were satisfied in the present data. Internal consistency was assessed using Cronbach’s alpha, with values between 0.70 and 0.95 considered acceptable for health questionnaire research; values below 0.70 are generally regarded as suboptimal, while values exceeding 0.95 may indicate item redundancy [38, 43, 44]. It should be noted that ordinal regression or beta‐regression (after appropriate rescaling) would represent a more statistically conservative alternative given the ordinal and ceiling‐affected nature of ACC_score; OLS was chosen here for interpretive simplicity and comparability with similar literature, and the satisfactory residual diagnostics provide empirical support for this choice. Bivariate associations between ACC_score and 13 hypothesized predictors (all six HBM constructs plus CAM_score, Motiv_score, Trust_Medic, Trust_Science, AE_total, BAR_I44, and BAR_I45) were examined using Spearman’s *ρ* with Bonferroni correction (adjusted *α* = 0.0038, *k* = 13). Bonferroni correction was selected as the primary approach because it controls the family‐wise error rate, which is more conservative and appropriate for an exploratory study where false positives are a primary concern. Benjamini–Hochberg (BH) false‐discovery rate correction is reported secondarily to assess sensitivity to the choice of correction. For the multivariate OLS regression models, no additional correction for multiple comparisons was applied to the regression coefficients. This reflects a deliberate and prespecified analytical choice: in a theory‐driven multivariate model where all predictors are included simultaneously to control for one another, each coefficient represents a conditional association adjusted for all other predictors, rather than an independent test of a separate hypothesis. Applying family‐wise correction to regression coefficients in this context would be overly conservative and inconsistent with standard practice in HBM‐based behavioral research [40, 41]. Readers are reminded that all multivariate results are explicitly framed as exploratory, and coefficients with *p* values near the conventional threshold (notably CAM attitudes, *p* = 0.028, and perceived susceptibility, *p* = 0.029) would not survive even a modest Bonferroni correction for nine predictors (adjusted *α* = 0.006); these should, therefore, be interpreted with particular caution. Effect sizes were interpreted as negligible (*ρ* < 0.10), small (0.10–0.29), medium (0.30–0.49), or large (≥ 0.50 [45]).

Multiple OLS regressions were conducted with ACC_score as the dependent variable. All six HBM constructs were included in every model to ensure comprehensive theoretical coverage and to prevent selective predictor omission. Two models were specified: M0 (primary; *k* = 9 predictors: all HBM constructs, CAM_score, Motiv_score, Trust_Medic, AE_total, BAR_comp) and M1 (exploratory sensitivity model motivated by BAR_comp *α* = 0.601, *k* = 12, with BAR_comp replaced by four individual barrier items). Trust_Science (I59) was retained in bivariate analyses to ensure comprehensive coverage of all measured constructs but was excluded from multivariate models a priori due to its conceptual and empirical overlap with CAM_score: both constructs reflect a generalized disposition toward nonconventional or science‐based health information and their coinclusion would have introduced redundant explanatory variance without adding theoretical precision. This decision was prespecified prior to model estimation. Standardized (*β*) and unstandardized (B) coefficients, along with standard errors (SEs) and 95% confidence intervals (CIs), are reported for all coefficients. Variance inflation factors (VIFs) were examined in JASP; all VIF values were below 3 (primary model M0: 1.11–2.52). Subgroup comparisons utilized Kruskal–Wallis and Mann–Whitney *U* tests; the rank‐biserial *r* effect size was calculated as *r* = 1 − (2U)/(n_1_n_2_).

The study sample of *n* = 71 was determined by convenience and available time rather than through a formal a priori power calculation, a recognized limitation [45, 46]. Post hoc power calculations were not computed, as such analyses have been shown to be methodologically uninformative [47]. The sample (*n* = 71) was adequate to detect large effects (*r* ≥ 0.35) but underpowered for small‐to‐medium effects (*r* ≤ 0.25); absence of a priori power calculation is acknowledged as a limitation [45]. The ratio of cases to predictors in M0 is 71:9, which simplifies to 7.9:1, falling short of the recommended 10:1 [48]. M1, at 71:12 = 5.9:1, is explicitly exploratory, and findings should be treated accordingly. The interpredictor Spearman correlation matrix for all model predictors was inspected to assess potential redundancy; findings should be interpreted accordingly.

## 3. Results

### 3.1. Sample Characteristics

The analytic sample included 71 adults (average age = 37.5 years, SD = 11.9, range 20–80) with self‐reported physician‐diagnosed IDA. The majority were female (*n* = 68, 95.8%). Educational achievement was notably high: 69.0% held postgraduate degrees and 22.5% held bachelor’s degrees, a profile that significantly exceeds the distribution in the Romanian general population, limiting generalizability (see Section 5 for comparison with national reference data). Monthly income varied widely, with 26.8% reporting ≥ 10,000 RON. The composite adverse effect burden score was 9.13 (SD = 7.38, median = 8.0, IQR: 2.0–15.5); 39.4% of the participants had modified or discontinued treatment due to adverse events. Full characteristics are shown in Table 1.

**TABLE 1:** Sociodemographic and clinical characteristics of the sample (*N* = 71).

Characteristic	*n*	%
Age (years)^a^	37.5 (11.9)	20–80
Sex		
Female	68	95.8
Male	3	4.2
Education		
Postgraduate (master’s/doctorate)	49	69.0
Bachelor’s degree	16	22.5
Partial higher education/vocational	4	5.6
Secondary education	2	2.8
Monthly household income (RON)		
≥ 10,000	19	26.8
7000–9999	11	15.5
5000–6999	14	19.7
3000–4999	13	18.3
1500–2999	4	5.6
< 1500	2	2.8
Prefer not to say	8	11.3
Health insurance (Yes)	60	84.5
AE_total score, *M* (SD) (range 0–36)	9.13 (7.38)	Mdn = 8.0
Treatment modification due to AEs		
None	43	60.6
Switched supplement type	10	14.1
Temporary discontinuation	7	9.9
Considered modification only	8	11.3
Complete discontinuation	3	4.2
Self‐reported adherence ≥ 81% of doses	51	71.8

*Note:* RON = Romanian leu; *M* = mean; Mdn = median. IDA status is based on participant self‐report of a prior clinician diagnosis; records were not independently verified.

Abbreviations: AE = adverse effects, SD = standard deviation.

^a^Reported as *M* (SD); range 20–80. Unless, otherwise, noted, remaining continuous variables are reported as *n* (%).

### 3.2. HBM Construct Descriptives and Informed Acceptance Profile

Descriptive statistics for all HBM constructs and the primary outcome are shown in Table 2. Perceived susceptibility was high (*M* = 4.61, SD = 0.75 on a 1–5 scale; 84.5% rated ≥ 4). Self‐efficacy was also elevated (*M* = 8.62, SD = 1.69 on a 2–10 scale, Mdn = 9.0). BAR_comp excludes Item 46 and shows *α* = 0.601 (suboptimal; see Section 2.2). The mean ACC_score was 20.68 (SD = 3.25, Mdn = 21, IQR: 19–23, range: 13–25). The IQR of only 4 points on a 20‐point scale suggests a substantial ceiling effect—the majority of participants scored in the upper quarter of the scale—which may weaken all observed bivariate and regression associations. Item‐level analysis revealed that willingness to accept if recommended by a physician (I33) had the highest mean score (*M* = 4.44, SD = 0.71; 90% positive responses), while willingness to participate in a clinical trial (I36) had the lowest (*M* = 3.82, SD = 1.17; 68% positive, 17% negative). Corrected item‐total correlations for ACC_score ranged from 0.46 (I36) to 0.71 (I32), indicating that all items contribute meaningfully to the overall measure.

**TABLE 2:** Descriptive statistics for HBM constructs and primary outcome (*N* = 71).

Variable	Range	*M*	SD	Mdn	IQR	*α*
HBM constructs						
HBM severity (HBM_sev)	2–10	5.77	1.99	6.0	4.0–7.0	—^b^
HBM susceptibility (HBM_sus)	1–5	4.61	0.75	5.0	4.0–5.0	—^c^
HBM benefits (HBM_ben)	1–5	3.76	0.93	4.0	3.0–4.0	—^c^
Self‐efficacy (HBM_se)	2–10	8.62	1.69	9.0	8.0–10.0	0.834
Barriers composite (BAR_comp)^a^	4–20	17.18	2.36	18.0	16.0–19.0	0.601^†^
Related constructs						
CAM attitudes (CAM_score)	5–25	20.15	4.72	21.0	17.0–24.0	0.838
Motivators (Motiv_score)	7–35	31.56	3.69	33.0	30.0–34.0	—
Trust in physician (I58)	1–5	4.13	0.88	4.0	4.0–5.0	—
Trust in science (I59)	1–5	4.32	0.86	5.0	4.0–5.0	—
Primary outcome						
ACC_score (informed acceptance)	5–25	20.68	3.25	21.0	19.0–23.0	0.801
I32: Willingness to try (*r*_it = 0.71)	1–5	4.28	0.74	4.0	4.0–5.0	—
I33: If physician recommends (*r*_it = 0.56)	1–5	4.44	0.71	5.0	4.0–5.0	—
I34: Within 6 months (*r*_it = 0.70)	1–5	4.06	0.79	4.0	4.0–5.0	—
I35: Recommend to others (*r*_it = 0.63)	1–5	4.08	0.86	4.0	4.0–5.0	—
I36: Clinical trial participation (*r*_it = 0.46)	1–5	3.82	1.17	4.0	3.0–5.0	—

*Note: M* = mean; Mdn = median; IQR = interquartile range; *α* = Cronbach’s alpha; *r*_it = corrected item‐total correlation. ACC_score was measured after provision of a standardized information text (Section 2.2). Range values in the table reflect the theoretical scale range for each instrument; observed ranges may differ (e.g., ACC_score observed range = 13–25, not 5–25).

Abbreviations: CAM = complementary and alternative medicine, SD = standard deviation.

^a^BAR_comp: I44, I45, I47, I48 only; Item 46 excluded (data error, see Section 2.2).

^b^HBM_sev: two‐item composite; Spearman–Brown reliability not reported due to scale range.

^c^HBM_sus and HBM_ben: single‐item measures; alpha not calculable; reliability uncertain.

^†^ *α* = 0.601 < 0.70 [38], indicating multidimensionality; items examined individually in M1 (Table 3).

### 3.3. Bivariate Associations With Acceptance

Spearman’s rank correlations with Bonferroni correction (*k* = 13, adjusted *α* = 0.0038) are shown in Table 3. Note that *k* was increased to 13 to include HBM_sus, a full HBM construct, which is appropriately part of a comprehensive HBM‐informed analysis. Five predictors demonstrated statistically significant associations with ACC_score after Bonferroni correction: self‐efficacy (*ρ* = 0.672, large effect), perceived benefits (*ρ* = 0.519), CAM attitudes (*ρ* = 0.500; both large), perceived susceptibility (*ρ* = 0.383), and motivators (*ρ* = 0.378; both medium; all *p*_Bonferroni ≤ 0.016). Trust in science nearly reached, but did not achieve, Bonferroni‐corrected significance (*ρ* = 0.331, *p*_Bonferroni = 0.062); under the BH correction, Trust_Science also met the 5% false‐discovery rate threshold (*p*_BH = 0.010); this finding is regarded as exploratory given the more conservative Bonferroni primary criterion. Current AE burden showed no meaningful association with ACC_score (*ρ* = −0.022, *p* = 0.856). Importantly, the ceiling effect in ACC_score (IQR = 19–23 on a 5–25 scale) likely attenuates all observed *ρ* values; the reported correlations should, therefore, be regarded as conservative estimates of the true population associations. The corresponding interpredictor heatmap, based on Spearman rank correlations among the model variables, is provided in Supporting Figure S4.

**TABLE 3:** Spearman rank correlations between predictors and ACC_score (*N* = 71, *k* = 13).

Predictor	*ρ*	*p* (uncorr.)	*p* (Bonferroni)	*p* (BH‐FDR)	Sig.	Effect
Self‐efficacy (HBM_se)	**0.672**	**< 0.001**	**< 0.001**	**< 0.001**	^**∗∗∗**^	**Large**
Perceived benefits (HBM_ben)	**0.519**	**< 0.001**	**< 0.001**	**< 0.001**	^**∗∗∗**^	**Large**
CAM attitudes (CAM_score)	**0.500**	**< 0.001**	**0.0001**	**< 0.001**	^**∗∗∗**^	**Large**
Susceptibility (HBM_sus)	**0.383**	**0.001**	**0.013**	**0.003**	^**∗**^	**Medium**
Motivators (Motiv_score)	**0.378**	**0.001**	**0.016**	**0.003**	^**∗**^	**Medium**
Trust in science (I59)	0.331	0.005	0.062	0.010	^†^	Medium
Trust in physician (I58)	0.183	0.127	1.000	0.184	ns	Small
Severity (HBM_sev)	0.185	0.123	1.000	0.184	ns	Small
BAR_I44: Safety concern	+0.288	0.015	0.195	0.028	^†^/BH^∗^	Small
BAR_I45: Lack of LT evidence	−0.115	0.341	1.000	0.443	ns	Small
BAR_comp	−0.107	0.374	1.000	0.443	ns	Negl.
BAR_I47: Efficacy uncertainty	−0.191	0.111	1.000	0.181	ns	Small
BAR_I48: Contamination risk	−0.078	0.518	1.000	0.562	ns	Negl.
AE burden (AE_total)	−0.022	0.856	1.000	0.856	ns	Negl.

*Note:* Bonferroni correction: adjusted *α* = 0.0038 (*k* = 13). BH‐FDR = Benjamini–Hochberg false‐discovery rate correction. Effect sizes (Cohen, 1988): negligible *ρ* < 0.10; small: 0.10–0.29; medium: 0.30–0.49; large: ≥ 0.50.

Abbreviation: ns = nonsignificant.

^∗∗∗^ *p*_Bonf < 0.001.

^∗^ *p*_Bonf < 0.05.

^†^ *p*_Bonf < 0.10.

^†^/BH^∗^ = not significant after Bonferroni but significant after BH‐FDR. BAR_I44 positive direction discussed in Section 4.3.

### 3.4. Multivariate Associations With Acceptance

OLS regression results for M0 and M1 are shown in Table 4. The main model (M0, *k* = 9, including all HBM constructs) explained 63.8% of the variance in ACC_score (*R* ^2^ = 0.638, *R* ^2^ adj = 0.585, *F* (9,61) = 11.97, *p* < 0.001; residuals were normal, SW *W* = 0.972, *p* = 0.110; see Supporting Figure S2 for the standardized residuals histogram and Supporting Figure S3 for the residuals versus predicted values plot). To assess the influence of the two single‐item HBM constructs on model fit, leave‐one‐out sensitivity analyses were conducted using the raw data: removing HBM_sus reduced *R* ^2^ from 0.638 to 0.609 (Δ*R* ^2^ = 0.030), while removing HBM_ben reduced *R* ^2^ by only 0.002 (*R* ^2^ = 0.637). Removing both simultaneously reduced *R* ^2^ to 0.600 (Δ*R* ^2^ = 0.038). These results confirm that HBM_sus contributes meaningfully to the model and that the collapse of HBM_ben from *ρ* = 0.519 (bivariate) to *β* = 0.055 (multivariate) reflects measurement noise attributable to its single‐item operationalization rather than genuine confounding, providing empirical support for the interpretation offered in Section 2.2. Four predictors had significant independent associations: self‐efficacy (*B* = 0.737, 95% CI [0.33, 1.15], *β* = 0.384, *p* < 0.001), perceived susceptibility (*B* = 0.992, 95% CI [0.11, 1.88], *β* = 0.229, *p* = 0.029), barriers composite (*B* = −0.378, 95% CI [−0.64, −0.12], *β* = −0.275, *p* = 0.005), and CAM attitudes (*B* = 0.170, 95% CI [0.02, 0.32], *β* = 0.248, *p* = 0.028). All other predictors were nonsignificant (all *p* > 0.24). All VIF values were below 2.6 (range 1.11–2.52). Multicollinearity diagnostics confirmed that no predictor exceeded acceptable thresholds: VIF values ranged from 1.11 to 2.52 in M0 and remained below 3.0 in M1, indicating that the regression estimates were not substantially influenced by inter‐predictor redundancy.

**TABLE 4:** OLS regression coefficients with 95% CI: primary model (M0) and sensitivity analysis (M1).

**Predictor**	**M0 (*k* = 9)** **R** ^2^ **= 0.638** ^**∗∗∗**^ **R** ^2^ **adj = 0.585 B**	**M1 (*k* = 12)** **R** ^2^ **= 0.666** ^**∗∗∗**^ **R** ^2^ **adj = 0.597 SE**	**β**	**p**	**95% CI**	**B**	**p**	**95% CI**

Intercept	**+6.681**	**2.674**	—	**0.015** ^**∗**^	**[1.34, 12.03]**	**+6.308**	**0.020** ^**∗**^	**[1.02, 11.60]**
Self‐efficacy (HBM_se)	**+0.737**	**0.206**	**0.384**	**< 0.001** ^**∗∗∗**^	**[0.33, 1.15]**	**+0.630**	**0.005** ^**∗∗**^	**[0.20, 1.06]**
HBM susceptibility (HBM_sus)	**+0.992**	**0.443**	**0.229**	**0.029** ^**∗**^	**[0.11, 1.88]**	**+0.979**	**0.030** ^**∗**^	**[0.10, 1.86]**
Barriers (BAR_comp)^a^	**−0.378**	**0.130**	**−0.275**	**0.005** ^**∗∗**^	**[−0.64, −0.12]**	—	—	—
I45: Lack of long‐term evidence	—	—	—	—	—	**−0.746**	**0.015** ^**∗**^	**[−1.34, −0.15]**
I47: Efficacy uncertainty	—	—	—	—	—	−0.561	0.097^†^	[−1.23, +0.10]
I44: Safety concern	—	—	—	—	—	+0.546	0.245ns	[−0.38, +1.48]
I48: Contamination risk	—	—	—	—	—	−0.303	0.439ns	[−1.08, +0.48]
Perceived benefits (HBM_ben)	+0.191	0.364	0.055	0.602ns	[−0.54, +0.92]	+0.126	0.732ns	[−0.61, +0.86]
CAM attitudes (CAM_score)	+0.170	0.076	0.248	0.028^∗^	[0.02, 0.32]	+0.162	0.044^∗^	[0.00, 0.32]
Motivators (Motiv_score)	+0.128	0.108	0.146	0.241ns	[−0.09, +0.34]	+0.108	0.319ns	[−0.11, +0.32]
Trust in physician (I58)	+0.200	0.312	0.054	0.524ns	[−0.42, +0.82]	+0.177	0.569ns	[−0.44, +0.80]
AE burden (AE_total)	−0.025	0.036	−0.057	0.489ns	[−0.10, +0.05]	−0.048	0.221ns	[−0.13, +0.03]
HBM severity (HBM_sev)	+0.136	0.149	0.084	0.366ns	[−0.16, +0.43]	+0.207	0.177ns	[−0.10, +0.51]
Model fit		*R* ^2^ adj = 0.585		*F* (9,61) = 11.97^∗∗∗^	SW resid *p* = 0.110		*F* (12,58) = 9.65^∗∗∗^	

*Note:* Dependent variable: ACC_score (*N* = 71). B = unstandardized coefficient; *β* = standardized coefficient, reported for M0 only; 95% CI = confidence interval. All VIF < 3 in M0 (range 1.11–2.52); all VIF < 3 in M1; Item 46 excluded (data error); *α* = 0.601. M1 is a prespecified exploratory sensitivity model motivated by BAR_comp’s multidimensionality (*α* = 0.601); n/k = 5.9, requiring exploratory interpretation of individual coefficients. Wide CI for I45 in M1 reflects coefficient instability at this n/k. — = predictor not in this model. Sensitivity analysis, including Item 46 as a binary covariate, confirmed a negligible impact on main coefficients (I46 *B* = −0.21, *p* = 0.697; Δ*R* ^2^ < 0.001).

Abbreviations: SE = standard error, ns = nonsignificant.

^a^BAR_comp = I44 + I45 + I47 + I48.

^†^ *p* < 0.10.

^∗∗∗^ *p* < 0.001.

^∗∗^ *p* < 0.01.

^∗^ *p* < 0.05.

The exploratory sensitivity model (M1, *k* = 12, with barriers broken down into individual items) produced *R* ^2^ = 0.666, *R* ^2^ adj = 0.597, *F* (12, 58) = 9.650, and *p* < 0.001, indicating statistical significance. The ratio of sample size to the number of predictors (n/k) was 5.9 in Model 1; therefore, all findings from this model should be viewed as exploratory and interpreted with caution. Self‐efficacy remained a significant predictor (*B* = 0.630, 95% CI [0.20, 1.06], *p* = 0.005) as did perceived susceptibility (*B* = 0.979, 95% CI [0.10, 1.86], *p* = 0.030). Attitudes toward CAM also reached significance (*B* = 0.162, 95% CI [0.00, 0.32], *p* = 0.044). Concerning barriers, only the lack of long‐term evidence (I45) showed a significant, independent, negative association (*B* = −0.746, 95% CI [−1.34, −0.15], *p* = 0.015), with the broad CI reflecting considerable coefficient instability at n/k = 5.9 and warranting cautious interpretation. Safety concerns (I44) continued to show a positive but nonsignificant trend (*B* = +0.546, 95% CI [−0.38, +1.48], *p* = 0.245), aligning with the pattern observed in the bivariate analysis.

### 3.5. Subgroup Analyses

Kruskal–Wallis tests found no statistically significant differences in ACC_score across education level (*H* = 0.210, df = 3, *p* = 0.976, *ε* ^2^ = 0.003), monthly household income (*H* = 4.966, df = 6, *p* = 0.548, *ε* ^2^ = 0.071; seven response categories including “Prefer not to say”), or AE burden tertile (*H* = 0.165, *p* = 0.921; low: *M* = 20.92; moderate: *M* = 20.36; high: *M* = 20.71). Mann–Whitney U found no statistically detectable difference by cost concern (*U* = 657.0, *p* = 0.258, rank‐biserial *r* = −0.165; *r* = 1 − [2 × 657]/(*n*_1_ × *n*_2_)). These null findings should not be interpreted as evidence of equivalence across subgroups. The study was underpowered for small between‐group effects (power < 73% for *ρ* ≤ 0.30), and the sample’s pronounced educational homogeneity (69.0% postgraduate) substantially reduces available between‐group variance. Furthermore, the ceiling effect in ACC_score (Section 3.2) may additionally constrain detectable subgroup differences. ACC_score showed no statistically detectable subgroup differences in this sample; whether this pattern holds in more representative populations requires investigation in larger, clinically verified samples.

## 4. Discussion

This cross‐sectional study provides an initial HBM‐informed examination of informed acceptance of microalgae‐based iron supplements among Romanian adults with self‐reported physician‐diagnosed IDA. The primary model (M0), which included all six HBM constructs to ensure theoretical completeness, identified self‐efficacy, perceived susceptibility, barriers composite, and CAM attitudes as significant independent correlates of ACC_score. No statistically significant variation in ACC_score was detected across sociodemographic and clinical subgroups although these null findings are subject to the power and ceiling‐effect constraints described in Section 3. These findings are exploratory and hypothesis‐generating.

### 4.1. Acceptance Profile: Context and the Ceiling Effect

The mean ACC_score of 20.68 (IQR 19–23 on a 5–25 scale) reflects informed acceptance measured after participants were exposed to standardized product information, including current evidence limitations. To contextualize this level, it is worth noting that comparable studies on CAM and nutraceutical acceptance in patient populations have generally reported high initial acceptance intention scores when products are framed within a clinical information context, though direct numerical comparisons are constrained by heterogeneity in instruments and populations [51]. In the specific context of microalgae‐based supplementation, Lacurezeanu et al. (2025) documented consistent improvements in hematological parameters following Spirulina and Chlorella supplementation in vivo, establishing biological plausibility for the intervention. However, the present study suggests that, at least in a self‐selected IDA population already familiar with oral iron supplementation, this biological rationale provides context for clinical investigation but does not directly predict patient attitudes. The present study, therefore, examines informed acceptance intention as a distinct empirical question [17]. The high mean ACC_score observed here is, therefore, interpretable as reflecting the convergence of perceived therapeutic relevance and product familiarity within a healthcare‐engaged sample, rather than as a generalizable population estimate. The pronounced ceiling effect, with only 9.9% scoring ≤ 15, is an important interpretive consideration. Because most participants scored near the maximum, the scale provides limited sensitivity to variation among higher‐acceptance respondents, and all observed correlations and regression coefficients are likely attenuated relative to their true population values. This should be borne in mind when interpreting both the magnitude and the statistical significance of all reported associations. A further consequence of the ceiling effect that warrants explicit acknowledgment is that the high *R* ^2^ = 0.638 in M0 should be interpreted with caution: restricted variance in a ceiling‐affected outcome can paradoxically inflate *R* ^2^ estimates and increase the risk of model overfitting relative to what adjusted *R* ^2^ shrinkage alone captures, particularly given the suboptimal case‐to‐predictor ratio of 7.9:1 in M0. No cross‐validation was conducted, and the difference between *R* ^2^ = 0.638 and *R* ^2^adj = 0.585 (Δ*R* ^2^ = 0.053) represents a nontrivial shrinkage estimate; the true population *R* ^2^ should be assumed lower than the sample estimate. As a complementary sensitivity check, we dichotomized ACC_score at the 75th percentile (≥ 23 = high acceptance; *n* = 24 vs. *n* = 47) and re‐estimated the primary model using binary logistic regression. Critically, both key effects were nonsignificant in this analysis: self‐efficacy (OR = 4.01, 95% CI [0.96, 16.81], *p* = 0.058) and CAM attitudes (OR = 3.12, 95% CI [0.71, 13.71], *p* = 0.133) showed directional consistency with the OLS findings but did not reach statistical significance. These nonsignificant results most likely reflect information loss from dichotomization and the small high‐acceptance group (*n* = 24), rather than a genuine absence of an effect. The reduced statistical precision of the logistic model (McFadden *R* ^2^ = 0.27) [52] relative to OLS reflects information loss from dichotomization and the smaller sample size in the high‐acceptance group, consistent with the ceiling‐effect interpretation. These results are regarded as exploratory and are reported to corroborate the directional consistency of the OLS findings rather than as independent confirmatory evidence. Future studies should use instruments with greater discriminative capacity, such as visual analog scales or expanded Likert formats, to reduce ceiling effects from the outset.

### 4.2. Self‐Efficacy and Perceived Susceptibility as Independent Correlates

Self‐efficacy showed the strongest independent association with ACC_score in both M0 (*β* = 0.384, *p* < 0.001) and M1 (*B* = 0.630, *p* = 0.005). This is consistent with theoretical expectations from the HBM and Social Cognitive Theory, which posit self‐efficacy as a key proximal determinant of health behavior intentions [53, 54]. Notably, HBM_sus is the single‐item construct whose removal from the model produces the largest reduction in fit (Δ*R* ^2^ = 0.030; see Section 3.4), suggesting it contributes meaningfully despite its single‐item limitation. Within the behavior‐change literature, self‐efficacy has been identified as a frequently important variable in HBM‐based studies, though effect sizes vary considerably across health domains and population contexts [55, 56], and the model has been applied as an explanatory framework in health communication research [57]. The magnitude of the self‐efficacy effect observed here (*β* = 0.384) is notable in the broader HBM literature. Carpenter’s (2010) meta‐analysis of 18 HBM studies identified perceived barriers (mean weighted *r* = −0.21) and perceived benefits as the strongest predictors of health behavior across diverse contexts; notably, self‐efficacy was not included as a separate construct in that meta‐analysis due to insufficient representation across the reviewed studies. The self‐efficacy effect observed here (*β* = 0.384), therefore, cannot be directly benchmarked against Carpenter’s findings, but it is consistent with the broader expectation that self‐efficacy is a proximal determinant of health behavior intentions. This may be attributable to the novelty of the intervention: microalgae‐based iron supplements may be unfamiliar to many IDA patients in Romania and lack the routine prescription context of conventional ferrous preparations. When a health product is novel, patients with lower perceived capability to evaluate, adopt, and manage it may be substantially more hesitant than in familiar supplementation contexts, amplifying the role of self‐efficacy as a discriminating factor. The sensory and esthetic characteristics of algae‐derived products, including taste, odor, and appearance, that differ markedly from conventional supplements, may represent a specific efficacy barrier that conventional HBM applications to medication adherence do not capture. These sensory dimensions cannot be captured by questionnaire‐based intention measures and constitute a recognized methodological boundary of the present study; future research should complement intention‐based surveys with sensory evaluation panels and taste‐acceptability trials to provide a complete acceptance profile. A specific psychometric consideration also warrants explicit discussion here. Both HBM_sus and HBM_ben were operationalized using single items, a pragmatic choice necessitated by the absence of validated, multi‐item Romanian‐language scales for these constructs in the IDA context. Single‐item measures are known to carry higher measurement error than multi‐item subscales, which can attenuate observed associations through random error and reduce the sensitivity of the instrument to detect true between‐person variation [34–36]. In practical terms, the collapse of HBM_ben from a large bivariate correlation (*ρ* = 0.519) to a nonsignificant multivariate coefficient (*β* = 0.055) is most parsimoniously attributable to this measurement limitation rather than to genuine confounding, a conclusion supported by the leave‐one‐out analysis showing that removing HBM_ben reduces *R* ^2^ by only 0.002. For HBM_sus, the single‐item constraint likely underestimates the true strength of the perceived susceptibility–acceptance relationship, though its significant contribution in both M0 and M1 suggests sufficient signal even under these conditions. Future studies should prioritize development or cultural adaptation of multi‐item, validated Romanian‐language HBM subscales for the IDA context to address this gap. Perceived susceptibility also showed a significant independent association (M0: *β* = 0.229, *p* = 0.029), consistent with the HBM proposition that perceived personal risk motivates openness to alternatives. Among patients who perceive their IDA as a personally relevant and ongoing threat, openness to a novel supplementation form appears greater, a pattern interpretable as motivated alternative‐seeking driven by perceived inadequacy of current treatment tolerance. Together, these two constructs suggest that both perceived personal relevance of IDA and confidence in one’s ability to use a novel supplement are potentially relevant targets for patient communication strategies; however, this interpretation requires prospective confirmation in clinically verified samples before any intervention recommendations can be drawn.

### 4.3. CAM Attitudes as an Independent Correlate of Acceptance

CAM attitudes (CAM_score; *β* = 0.248, *p* = 0.028 in M0) emerged as the fourth significant independent predictor of acceptance. Patients with more positive prior orientations toward complementary and alternative medicine reported greater informed acceptance of microalgae‐based iron supplements, independent of their HBM beliefs and current adverse‐effect burden. This is consistent with established evidence that prior CAM attitudes represent a stable cognitive schema that shapes willingness to consider nonconventional therapeutic options [51, 58]. In the present context, microalgae‐derived iron can be conceptualized as occupying an intermediate position between conventional supplementation and integrative nutrition: it is derived from a natural biological source and embedded in a nutritional matrix, which may render it more congruent with the health philosophies of patients who are already positively oriented toward CAM. The finding also has a specific clinical implication: in a heterogeneous IDA population, patients with more negative CAM attitudes may require different communication strategies, specifically, greater emphasis on the evidence base and clinical validation of microalgae‐based preparations compared with patients already favorably disposed toward integrative approaches. The CAM attitudes effect did not reach significance in the binary logistic sensitivity analysis (OR = 3.12, *p* = 0.133), which likely reflects a loss of statistical power from dichotomizing the outcome rather than genuine effect absence; the directional consistency across both models supports treating this finding as a meaningful exploratory signal. Replication with validated CAM attitude scales and in more representative samples is needed before clinical translation.

### 4.4. Exploratory Observations Regarding safety Concerns and Evidence‐Related Barriers

The positive bivariate association between safety concern (I44) and ACC_score (*ρ* = +0.288, *p*_Bonf = 0.195) is counterintuitive and warrants careful interpretation. Before substantive interpretation, the coding direction of I44 was verified: the item was administered as an affirmatively worded statement reflecting the presence of safety concern (higher score = greater concern), and no reverse coding was applied or required. The positive association, therefore, reflects a genuine pattern in the data rather than a scoring artifact. One plausible substantive explanation is that patients who are actively attentive to the side effect profile of their current treatment are simultaneously more motivated to explore alternatives they perceive as better tolerated, a pattern consistent with the “health‐engaged patient” phenomenon described in patient activation literature, wherein heightened health monitoring co‐occurs with proactive information‐seeking and openness to therapeutic alternatives [59]. Under this interpretation, safety concerns operate not as a deterrent but as a motivator of alternative‐seeking among patients already engaged in their condition management. However, several methodological alternative explanations must be considered before any substantive interpretation is adopted. First, acquiescence bias, the tendency of respondents on Likert scales to endorse items regardless of content, may inflate co‐occurrence between positively worded items across scales, particularly in a self‐administered online format without interviewer presence. Second, I44 is a single‐item measure of a multidimensional construct (safety monitoring); its distributional properties, framing, and relationship to the broader barriers composite (in which it did not load consistently, *α* = 0.601) introduce substantial measurement uncertainty. Third, the association did not survive Bonferroni correction and was nonsignificant in M1 (*B* = +0.546, *p* = 0.245), indicating that the signal is at best marginal and may not replicate. At most, this finding tentatively suggests that safety concerns about current iron formulations do not necessarily predict rejection of alternative products, but this requires replication with multi‐item, validated instruments before any communication or clinical implications can be responsibly inferred [34].

### 4.5. Lack of Long‐term Evidence as the Most Salient Evidence‐Related Barrier

In the sensitivity model M1, the lack of long‐term research evidence (I45) was the only individual barrier with a significant, independent, negative association (*B* = −0.746, 95% CI [−1.34, −0.15], *p* = 0.015). The wide CI reflects substantial coefficient instability at n/k = 5.9 and signals that the point estimate should be treated as an approximation rather than a precise effect size. Nonetheless, the direction is mechanically plausible and consistent with a well‐established pattern in CAM and nutraceutical acceptance research: patients who apply evidence‐based criteria to therapeutic decisions are more likely to reserve acceptance for interventions supported by robust, long‐term clinical data [51]. For microalgae‐based iron supplementation specifically, the available clinical evidence base, while promising, as documented in recent systematic reviews including Lacurezeanu et al. remains predominantly derived from short‐duration in vivo studies and lacks large‐scale, long‐term randomized controlled trials that clinicians and informed patients typically use as benchmarks for confidence [17]. This gap between emerging biological evidence and the scale of clinical validation expected by evidence‐oriented patients may be an actionable target: research investments in multicenter, longer‐duration RCTs examining hematological outcomes, tolerability, and safety over 12–24 months would not only advance the evidence base but, if findings replicate, could directly address the barrier identified by I45 in this sample. The present data alone are insufficient to recommend specific communication strategies, but they suggest that, for evidence‐oriented patient subgroups, providing a transparent, appropriately caveated summary of the current state of clinical evidence, explicitly acknowledging what is and is not yet known, may be more persuasive than presenting only positive short‐term findings.

### 4.6. Physician Recommendation as a Potentially Relevant Contextual Factor

Item I33, which measured acceptance conditional on a physician’s recommendation, emerged as the highest‐scoring acceptance item (*M* = 4.44/5; 90% positive). It should be noted that Item I33 measured conditional acceptance contingent on a hypothetical physician recommendation, and we are not aware of evidence that microalgae‐based iron preparations are currently recommended by physicians as part of standard IDA management protocols; the clinical translation of this finding, therefore, remains prospective. This is consistent with evidence indicating that provider‐specific endorsement can play a substantial role in shaping patient acceptance, intention to use, and uptake of CAM, nutraceutical, and other health‐related innovations [51]. In contrast, the absence of a significant association for generalized physician trust (I58; *p* = 0.524 in M0) suggests that the observed effect may be attributable less to a broad trust orientation and more to the influence of concrete, case‐specific professional recommendations. This interpretation is consistent with the literature, which shows that clinician endorsement may operate through tailored communication, reassurance, and benefit–risk framing, thereby influencing acceptance beyond baseline trust alone [60].

Because I33 is a component item of the ACC_score dependent variable rather than an independent predictor, no causal or mechanistic inference can be drawn from its item‐level distribution. The notably high mean for I33 is reported descriptively and should motivate future studies designed specifically to examine the provider‐recommendation pathway using appropriate prospective designs.

### 4.7. AE Burden and Informed Acceptance: A Null Finding

The near‐zero bivariate correlation between AE burden and ACC_score (*ρ* = −0.022, *p* = 0.856) was corroborated by the absence of statistically detectable differences across AE tertiles (*H* = 0.165, *p* = 0.921). Two competing interpretations of this null finding should be explicitly considered before any substantive conclusion is drawn. The first, substantive interpretation is that cognitive and belief‐based factors such as self‐efficacy, perceived susceptibility, and CAM attitudes may be more proximal correlates of acceptance intention than current somatic experience. However, given the ceiling effect and small sample size, the absence of a detectable AE burden association should not be interpreted as evidence that no such relationship exists. Under this interpretation, AE burden may function more as a contextual background variable that motivates product‐seeking in general (i.e., contributing to why patients are open to alternatives at all) rather than as a discriminating predictor of which patients within a motivated sample are more or less open. The second methodological interpretation is that the ceiling effect in ACC_score (IQR = 19–23 on a 5–25 scale) compresses outcome variability so severely that no predictor, including AE burden, could be expected to yield a detectable association regardless of the true underlying relationship. This methodological explanation is at least as parsimonious as the substantive one and cannot be excluded on the basis of the present data. The two interpretations carry different implications. If the first is correct, AE burden is genuinely not a useful stratification variable for acceptance of microalgae supplementation. If the second is correct, the relationship remains an open empirical question requiring instruments with higher ceiling tolerance. Future studies should address this directly by using outcome measures with greater discriminative capacity and by recruiting samples with more heterogeneous acceptance profiles, including patients who have explicitly refused or discontinued iron supplementation.

### 4.8. Clinical Implications

Given the exploratory nature of this study and its substantial methodological limitations, clinical recommendations cannot be drawn from these data alone. Nonetheless, the pattern of findings suggests several hypothesis‐generating directions that may be relevant for clinicians working in integrative nutrition and complementary medicine settings, pending replication. First, the central role of self‐efficacy (*β* = 0.384) suggests that patient confidence in managing a novel supplementation regimen may be a more proximal determinant of acceptance intention than current adverse effect experience. If this pattern replicates prospectively, it would support incorporating brief, targeted self‐efficacy‐enhancing communication into consultations in which microalgae‐based or other novel iron preparations are being considered, for example, by providing concrete, practical information about product preparation, dosing, and what to expect, rather than focusing primarily on evidence summaries. This approach is consistent with self‐efficacy‐informed patient education principles derived from Social Cognitive Theory [53] and has demonstrated effectiveness in improving adherence intentions across analogous supplementation contexts. Second, the independent contribution of CAM attitudes to acceptance (*β* = 0.248) implies that patients’ prior orientation toward complementary medicine is a relevant stratification variable in clinical practice. Clinicians may benefit from briefly assessing patients’ general attitudes toward CAM as part of shared decision‐making conversations about iron supplementation alternatives, as this information could inform the framing and depth of evidence communication. Third, the signal from I45 that lack of long‐term evidence is a salient barrier for some patients suggests that clinicians should proactively and transparently address the current state of the evidence base for microalgae‐based iron preparations, including honest acknowledgment of what remains unknown, rather than framing the product solely in terms of existing positive findings. Finally, the high proportion of participants endorsing conditional acceptance depending on physician recommendation (I33; *M* = 4.44/5) reinforces the established importance of clinician endorsement in CAM uptake. These implications are tentative and hypothesis‐generating; they should not be operationalized in clinical practice without prospective confirmation in larger, clinically verified, and more representative IDA populations.

## 5. Limitations

Validation studies across chronic conditions show that self‐reported diagnoses can differ from clinically confirmed case definitions [61–64]; IDA‐specific validation data remain scarce, as IDA requires laboratory confirmation for definitive classification. Second, the use of online convenience sampling significantly limits external validity. The sample was highly educated (91.5% university level and 69.0% postgraduate) and predominantly female (95.8%), a profile that departs substantially from the Romanian adult population (Table 5), where postgraduate degree attainment is approximately 10%–12% and is likely to underrepresent individuals with lower levels of education, limited digital access, or greater reliance on public‐sector care. Methodological research on nonprobability samples shows that these recruitment methods are prone to selection bias and limit the ability to draw population‐level conclusions unless supported by probability‐based sampling or strong adjustment techniques [65]. Therefore, these findings should be considered specific to adult women with self‐reported physician‐diagnosed IDA recruited through an online Romanian convenience sample, rather than widely applicable to all adults with IDA. In accordance with SAGER guidelines [66], the near‐exclusively female composition of this sample (95.8% female) constitutes a sex‐related limitation: findings cannot be assumed to generalize to men or individuals of other genders, and sex‐disaggregated analyses were not feasible given the available sample size. As an additional sensitivity check, all primary OLS regression results were verified to remain substantively unchanged when the three male participants were excluded from the analysis (*n* = 68); the pattern of significant predictors and the direction and approximate magnitude of all coefficients were consistent with the full‐sample results, indicating that the three male participants did not materially influence the findings.

**TABLE 5:** Comparison of study sample characteristics with Romanian population reference data.

Characteristic	Study sample (*N* = 71)	Romanian adult population	Reference
Female sex	95.8% (*n* = 68)	51.5%	[49]
University‐level education or higher (bachelor’s degree and above)	91.5% (*n* = 65)	16.0%	[50]
Postgraduate degree (master’s/doctorate)	69.0% (*n* = 49)	Approx. 10%–12%^∗^	[49]
Monthly household income ≥ 10,000 RON	26.8% (*n* = 19)	No directly comparable official proportion identified in the cited national summary statistics	[49]
Monthly household income ≥ 5000 RON	62.0% (*n* = 44)	National average household income: 6464 RON/month	[49]

*Note:* RPL 2021 = Recensământul Populației şi Locuințelor 2021, definitive results, published by the Romanian National Institute of Statistics (INS). Postgraduate attainment (10%–12%) is approximated from INS definitive census tables (Tables 3.04–3.05) for master’s and doctoral degree holders combined, expressed as a proportion of the population aged 25**+** [50].

Another limitation is that the IDA status depended solely on a self‐reported previous clinician’s diagnosis and was not independently verified. Validation studies across various health conditions show that self‐reported diagnoses can differ from clinically confirmed or registry‐based case definitions, with the level of agreement varying by condition and context; therefore, some misclassification of anemia status or subtype might occur in this study. It is important to note that validation studies specifically examining the accuracy of self‐reported IDA diagnosis are limited in the literature; the references cited here concern other conditions (depression, retirement‐related health reporting, and anxiety) and may not fully apply to the classification of IDA, which requires laboratory confirmation. Future studies should incorporate clinically verified IDA status to address this gap [61–64]. Additionally, measurement limitations impact several constructs. The barriers composite showed only moderate internal consistency (*α* = 0.601), indicating possible multidimensionality, while perceived susceptibility and perceived benefits were measured with a single item each, and perceived severity with just two items. Psychometric research indicates that measures with few items and low reliability increase measurement error and tend to weaken observed associations, suggesting that the coefficients for these HBM constructs may underestimate their actual relationships with acceptance [44]. The unusual binary format of Item 46 is another measurement irregularity although sensitivity analyses suggest that excluding it had little impact on the main results.

The interpretation of effect sizes is also limited by the distributional and design‐related features of the data. The acceptance score showed evidence of a ceiling effect, with most participants reporting relatively high levels of acceptance. Restricted outcome variability can weaken correlations and regression coefficients and may hide subgroup differences, especially in self‐report studies where favorable responding and social desirability may be heightened [37, 67]. Furthermore, all variables were measured using the same self‐administered questionnaire at a single time point, which raises the potential for common‐method variance. Although the Harman single‐factor test [67] provided some reassurance in this dataset, however, the specific variance accounted for by the first unrotated factor was not retained for reporting, which limits the falsifiability of this claim. Methodological literature warns that the Harman single‐factor test is widely regarded as an insufficient safeguard against common‐method variance [67], and the absence of a reported value means this reassurance cannot be independently verified. Readers should, therefore, treat common‐method bias as an unresolved concern, and using triangulation with objective or externally validated data is preferable in future studies. The small sample size, lack of an a priori power calculation, and suboptimal predictor‐to‐case ratio further limit interpretability, especially for null results and subgroup comparisons, which should be viewed as inconclusive rather than as definitive evidence of no association [65]. Finally, the outcome assessed reflected acceptance intention rather than actual adoption. Since intention and behavior do not always align, high acceptance scores should not be assumed to directly translate into real‐world adoption; future research should, therefore, explore whether these beliefs and barriers predict subsequent behavior in clinical or community settings [68, 69]. Additionally, the present study focused on belief‐based and attitudinal determinants of acceptance and did not assess product‐attribute preferences such as formulation format (e.g., capsule versus powder versus food integrated), dosage volume, or willingness to pay. These factors are likely to influence real‐world adoption and should be incorporated into future discrete‐choice or conjoint‐analysis studies of microalgae‐based iron supplementation. The questionnaire design further precluded the assessment of sensory or hedonic acceptance (taste, odor, and texture), which are important determinants of actual product uptake for microalgae‐based preparations and should be evaluated in dedicated sensory evaluation studies. Finally, prior familiarity with or previous consumption of microalgae products was not assessed. Participants who had previously used Spirulina or Chlorella supplements may have approached the standardized information text with pre‐existing frames of reference, potentially anchoring their acceptance scores to prior experience rather than evaluating the novel information de novo. Future studies should include validated items assessing prior microalgae awareness and consumption history as covariates in the acceptance model.

## 6. Conclusions

This study offers an initial HBM‐informed examination of informed acceptance of microalgae‐based iron supplements among Romanian adults with self‐reported, physician‐diagnosed IDA, to our knowledge the first such investigation conducted in a Romanian convenience sample of adults with self‐reported IDA. In this convenience sample, acceptance scores following standardized information provision were high and exhibited a pronounced ceiling effect, with four constructs: self‐efficacy, perceived susceptibility, perceived barriers, and CAM attitudes, emerging as significant independent predictors in the primary regression model. No statistically significant subgroup differences were detected, though this null finding is substantially constrained by limited statistical power and the ceiling effect in the outcome measure. Given the documented limitations of single‐survey designs, common‐method bias cannot be ruled out, and the Harman single‐factor test applied during analysis is acknowledged as an insufficient safeguard against common‐method variance. The operationalization of two HBM constructs using single items likely reduced the observed effect sizes. Self‐efficacy showed the strongest association in the primary OLS model though this result did not survive binary logistic sensitivity analysis, underscoring its tentative status; the hypothesis that self‐efficacy‐enhancing communication may be a relevant target in patient consultations warrants prospective testing but cannot be treated as established. These findings should be considered exploratory and hypothesis‐generating rather than definitive. Larger studies with clinically verified IDA samples, validated multi‐item HBM instruments, outcome measures with higher ceiling tolerance, and behavioral follow‐up are needed before any concrete clinical recommendations can be made.

## Author Contributions

Alexandra Lacurezeanu: conceptualization, data curation, formal analysis, investigation, methodology, writing–original draft, and writing–review and editing. Dan Cristian Vodnar: conceptualization, funding acquisition, resources, supervision, and writing–review and editing. Dan Cristian Vodnar had full access to all data in this study and takes full responsibility for the data’s integrity and the accuracy of the analysis.

## Funding

This work was supported by the Unitatea Executivă pentru Finanțarea Învățământului Superior, a Cercetării, Dezvoltării şi Inovării (UEFISCDI), Romania, grant number 14PCE, PN‐IV‐P1‐PCE‐2023‐1449.

## Disclosure

The funder had no role in study design, data collection, data analysis, data interpretation, manuscript preparation, or the decision to submit the article for publication. All authors have read and approved the final version of the manuscript.

## Ethics Statement

The study was approved by the Bioethics Committee of the University of Agricultural Sciences and Veterinary Medicine Cluj‐Napoca (Decision No. 535/24.10.2025; application no. 522/20.10.2025), in compliance with the Declaration of Helsinki. All participants provided electronic informed consent before participation.

## Consent

Please see the Ethics Statement.

## Conflicts of Interest

The authors declare no conflicts of interest.

## Supporting Information

Additional supporting information can be found online in the Supporting Information section.

## Supporting information


**Supporting Information 1** Supporting Information S1. Questionnaire used for data collection in the cross‐sectional study assessing the acceptance of microalgae‐based iron supplements among Romanian adults with self‐reported iron‐deficiency anemia.

**Supporting Information 2** The online version includes supporting information, including the completed STROBE checklist and Supporting figures. Supporting Figure S1. Participant flow diagram showing eligibility assessment, exclusions, and final analytic sample. Supporting Figure S2. Histogram of standardized residuals for the primary OLS regression model. Supporting Figure S3. Residuals versus predicted values plot for the primary OLS regression model. Supporting Figure S4. Spearman interpredictor correlation heatmap including ACC_score, Health Belief Model constructs, CAM attitudes, motivators, trust variables, barriers, adverse‐effect burden, age, and perceived impact of adverse effects.

## Data Availability

The anonymized dataset generated and analyzed during the current study is available from the corresponding author on reasonable written request, subject to institutional ethics committee approval for data sharing.
