# Supplementary material for: Factors Influencing Acceptance of Microalgae‐Based Iron Supplements in Romanian Adults With Self‐Reported Iron‐Deficiency Anemia: A Cross‐Sectional Study
Source: Anemia. 2026 Sep 8;2026:3201387. doi: 10.1155/anem/3201387 (PMC13551349; doi:10.1155/anem/3201387)
Supplement: Supplementary file 1 [file ANEM-2026-3201387-s001.docx]

|  | **Responses received via online survey platform**  **N = 72** | | |
| --- | --- | --- | --- |
| **↓** | | |  |
|  | **Assessed for eligibility criteria:**  (1) Age ≥ 18 years (2) Self-reported physician-diagnosed IDA (3) Current or prior oral iron supplementation | | |
| **↓** | | |  |
|  | **Excluded (n = 1)**  • Age < 18 years (n = 1)  • Did not meet IDA criteria: n = 0  • No supplementation history: n = 0  • Did not consent: n = 0  • Missing data on key variables: n = 0 | **Met all eligibility criteria**  **n = 71**  All 71 participants provided complete  data on all analysis variables  (complete-case rate = 100%) | |
| **↓** | | |  |
|  | **Included in primary analysis**  **n = 71**  Cross-sectional HBM-informed survey \| Romania, 2026 | | |

**Note.** Recruitment was conducted via convenience sampling through patient communities, online groups, and healthcare professional referral channels in Romania. The total number of individuals who encountered the survey link was not recorded; therefore, a response rate could not be calculated. The single exclusion was due to age < 18 years. IDA status was based on self-reported physician-confirmed diagnosis; clinical records were not independently verified. No data imputation was required (complete-case rate = 100%).

**Supplementary Figure S1.** *Participant flow diagram showing eligibility assessment, exclusions, and final analytic sample.*

**Supplementary** **Figure S2.** Histogram of standardized residuals for the primary OLS regression model.

**Supplementary Figure S3.** Residuals versus predicted values plot for the primary OLS regression model.

**Supplementary Figure S4.** Spearman inter-predictor correlation heatmap including ACC_score, Health Belief Model constructs, CAM attitudes, motivators, trust variables, barriers, adverse-effect burden, age, and perceived impact of adverse effects.
