# Supplementary material for: Factors Influencing Acceptance of Microalgae‐Based Iron Supplements in Romanian Adults With Self‐Reported Iron‐Deficiency Anemia: A Cross‐Sectional Study
Source: Anemia. 2026 Sep 8;2026:3201387. doi: 10.1155/anem/3201387 (PMC13551349; doi:10.1155/anem/3201387)
Supplement: Supplementary file 2 [file ANEM-2026-3201387-s002.docx]

**Patient Acceptance of Alternative Iron Supplements: A Survey on Microalgae-Based Treatment Options for Iron Deficiency Anemia**

Estimated time: 12–15 minutes

**Informed consent & data protection**

**Study information**

**Purpose:** This survey explores your experience with iron supplements and your interest in new alternatives. Your honest answers will help improve treatment options for patients with anemia.

**Conducted by:** Faculty of Food Science and Technology, University of Agricultural Sciences and Veterinary Medicine Cluj-Napoca

**Principal investigator:** Alexandra Lacurezeanu, Dan Cristian Vodnar

**Contact:** [alexandra.lacurezeanu@student.usamvcluj.ro](mailto:alexandra.lacurezeanu@student.usamvcluj.ro) , dan.vodnar@usamvcluj.ro

**Duration:** Approximately 12–15 minutes

**Your rights & data protection (GDPR)**

**Voluntary participation:** Your participation is completely voluntary. You may withdraw at any time without consequence.

**Data collection & use:**

We collect health information about your anemia history, treatment experience, and opinions.

Data will be used only for research purposes to improve anemia treatment options.

Your responses will be analyzed in aggregate with other participants.

**Confidentiality:**

Your responses are anonymous: we do not collect names or identifying information.

Data will be stored securely on encrypted servers.

Only authorized research team members will access the data.

Results will be published in aggregate form only.

**Data retention:** Survey data will be retained for 5 years as required by research standards, then securely deleted.

**Your rights:** Under GDPR, you have the right to: access your data (within 30 days of request); request deletion of your data; withdraw consent at any time; lodge a complaint with your national data protection authority.

**Data protection officer:** [alexandra.lacurezeanu@student.usamvcluj.ro](mailto:alexandra.lacurezeanu@student.usamvcluj.ro) and [dan.vodnar@usamvcluj.ro](mailto:dan.vodnar@usamvcluj.ro)

☐ **I have read and understood the above information**

☐ **I voluntarily consent to participate in this survey**

☐ **I understand my data will be processed as described above**

*You must check all boxes to proceed with the survey.*

**Section 1: about you**

**1.** Age: _____ years

**2.** Gender:

Male

☐ Female

☐ Non-binary

☐ Other

☐ Prefer not to say

**3.** Education level:

High school or less

☐ Some college / Technical training

☐ Bachelor's degree

☐ Graduate degree (master's/doctorate)

**4.** Monthly household income (net, after taxes):

Less than 1,500 lei

☐ 1,500–2,999 lei

☐ 3,000–4,999 lei

☐ 5,000–6,999 lei

☐ 7,000–9,999 lei

☐ 10,000 lei or more

☐ Prefer not to say

**5.** Do you have health insurance?

Yes

☐ No

**Section 2: your anemia history**

**6.** When were you diagnosed with iron deficiency anemia?

Less than 6 months ago

☐ 6 months to 1 year ago

☐ 1–2 years ago

☐ 2–5 years ago

☐ More than 5 years ago

**7. PRIMARY** cause of your anemia?

Heavy menstrual bleeding

☐ Pregnancy / postpartum

☐ Poor diet (low animal-source foods)

☐ Digestive problems (Crohn's, celiac disease, etc.)

☐ Surgery or blood loss

☐ Don't know

☐ Other: ____________

**8.** Have you ever needed: (check all that apply)

Oral iron pills / supplements

☐ IV iron infusion

☐ Blood transfusion

☐ None of the above

**9.** How many different oral iron supplements have you tried?

☐ None	☐ 1 type	☐ 2 types	☐ 3 types	☐ 4+ types

**10.** Are you currently taking iron supplements?

Yes

☐ No, but I took them before

☐ No, never tried them

*If you selected 'No, never tried them', skip to Question 17.*

**11.** How long have you taken iron supplements in total?

Less than 1 month

☐ 1–3 months

☐ 3–6 months

☐ 6–12 months

☐ More than 1 year

**Section 3: side effects from iron supplements**

*Think about the period when you were taking iron supplements. Rate how severe each problem was:*

**Scale:** 0 = None | 1–2 = Mild | 3–4 = Moderate | 5–6 = Severe

**12. Nausea or upset stomach:** ☐ 0 ☐ 1 ☐ 2 ☐ 3 ☐ 4 ☐ 5 ☐ 6

**13. Constipation:** ☐ 0 ☐ 1 ☐ 2 ☐ 3 ☐ 4 ☐ 5 ☐ 6

**14. Diarrhea:** ☐ 0 ☐ 1 ☐ 2 ☐ 3 ☐ 4 ☐ 5 ☐ 6

**15. Stomach pain or cramping:** ☐ 0 ☐ 1 ☐ 2 ☐ 3 ☐ 4 ☐ 5 ☐ 6

**16. Bloating:** ☐ 0 ☐ 1 ☐ 2 ☐ 3 ☐ 4 ☐ 5 ☐ 6

**17. Metallic taste:** ☐ 0 ☐ 1 ☐ 2 ☐ 3 ☐ 4 ☐ 5 ☐ 6

**18.** How much did side effects interfere with your daily life?

Not at all

☐ A little

☐ Somewhat

☐ Quite a bit

☐ Extremely

**19.** Have you ever stopped or reduced iron supplements because of side effects?

Yes, stopped completely

☐ Yes, stopped temporarily then restarted

☐ Yes, switched to a different type

☐ No

☐ Considered it but did not stop

**Section 4: treatment adherence**

**20.** Do you sometimes forget to take your iron supplement?

**21.** Have you stopped taking it without telling your doctor because it made you feel worse?

**22.** On average, what percentage of your prescribed doses do you actually take?

0–20%

☐ 21–40%

☐ 41–60%

☐ 61–80%

☐ 81–100%

**Section 5: your diet and lifestyle**

**23.** Which best describes your diet?

Omnivore (eat all foods)

☐ Vegetarian (no meat)

☐ Vegan (no animal products)

☐ Mostly plant-based

☐ Other: ____________

**24.** Do you currently take any other supplements? (check all that apply)

Multivitamins

☐ Vitamin D

☐ B vitamins

☐ Calcium

☐ Omega-3 / Fish oil

☐ Spirulina or Chlorella

☐ Other: ____________

☐ None

**Section 6: your health**

**25.** Do you have any of these conditions? (check all that apply)

Celiac disease or inflammatory bowel disease

☐ Kidney disease

☐ Diabetes

☐ Thyroid disorder

☐ Gastric bypass surgery

☐ None

☐ Other: ____________

**26. 26.** How much does anemia affect your daily life?

Not at all

☐ A little

☐ Moderately

☐ Quite a bit

☐ Extremely

**Section 7: your attitudes toward treatment**

**Rate your agreement:** 1 = Strongly disagree | 5 = Strongly agree

**27. I prefer natural treatments when available.**

☐ 1 ☐ 2 ☐ 3 ☐ 4 ☐ 5

**28. I believe natural remedies have fewer side effects than pharmaceutical drugs.**

☐ 1 ☐ 2 ☐ 3 ☐ 4 ☐ 5

**29. I trust plant-based supplements more than synthetic ones.**

☐ 1 ☐ 2 ☐ 3 ☐ 4 ☐ 5

**30. I am open to trying alternative treatments.**

☐ 1 ☐ 2 ☐ 3 ☐ 4 ☐ 5

**31. I believe in combining conventional and alternative medicine.**

☐ 1 ☐ 2 ☐ 3 ☐ 4 ☐ 5

**Section 8: about microalgae iron supplements**

**Please read carefully before answering the following questions.**

**Current challenges with iron supplements:**

Conventional iron tablets, such as ferrous sulfate or ferrous fumarate, are the standard treatment for iron deficiency anemia. However, **40–50% of patients experience gastrointestinal (GI) discomfort** such as nausea, abdominal cramps, or constipation. These side effects often lead to poor adherence, with less than 50% of patients continuing treatment as prescribed. In addition, iron absorption can be inhibited by certain dietary compounds, such as phytates, polyphenols, and calcium, which form insoluble complexes or compete for intestinal uptake sites.

**Why scientists are studying microalgae:**

Microalgae are microscopic aquatic organisms rich in nutrients, and some species have exceptionally high iron content and natural compounds that may improve absorption and reduce side effects.

Examples of promising species with established research evidence and authorized novel food status in the EU:

- ***Chlorella vulgaris***: contains approximately **419 mg of iron per 100 g**, along with chlorophyll, which may enhance iron uptake.
- ***Arthrospira platensis* (Spirulina)**: provides approximately **134 mg of iron per 100 g**, along with phycocyanin, an antioxidant pigment associated with improved tolerance and anti-inflammatory effects.
- ***Galdieria sulphuraria***: an extremophilic red alga with a **very high total iron content** and excellent potential for bioavailability.

**Why this research matters:**

Researchers are exploring these microalgae as natural, eco-friendly, and potentially better-tolerated alternatives to synthetic iron salts. Their combination may provide a sustainable iron source with fewer GI side effects, improved absorption, and lower environmental impact.

The following questions ask about your opinions, awareness, and willingness to use microalgae-based iron supplements as a possible alternative to traditional iron tablets.

**Section 9: your interest in trying a new alternative**

**32.** After reading the above, how willing are you to try microalgae iron supplements?

Definitely unwilling

☐ Probably unwilling

☐ Neutral / Unsure

☐ Probably willing

☐ Definitely willing

**33.** If your doctor/dietitian recommended this supplement, would you choose microalgae-based iron supplementation?

Definitely no

☐ Probably no

☐ Unsure

☐ Probably yes

☐ Definitely yes

**34.** How likely are you to try this in the next 6 months if it becomes available?

Extremely unlikely

☐ Unlikely

☐ Neither likely nor unlikely

☐ Likely

☐ Extremely likely

**35.** Would you recommend it to other anemia patients?

Definitely not

☐ Probably not

☐ Unsure

☐ Probably yes

☐ Definitely yes

**36.** Would you join a clinical trial testing this supplement?

Definitely not

☐ Probably not

☐ Unsure

☐ Probably yes

☐ Definitely yes

**Section 10: what would influence you?**

**Rate how important each factor is in your decision to try microalgae supplements:**

**Scale:** 1 = Not important | 5 = Extremely important

**37. Fewer stomach side effects:** ☐ 1 ☐ 2 ☐ 3 ☐ 4 ☐ 5

**38. Natural / plant-based origin:** ☐ 1 ☐ 2 ☐ 3 ☐ 4 ☐ 5

**39. Better iron absorption:** ☐ 1 ☐ 2 ☐ 3 ☐ 4 ☐ 5

**40. Additional vitamins and nutrients (B12, C, folate):** ☐ 1 ☐ 2 ☐ 3 ☐ 4 ☐ 5

**41. Environmentally sustainable:** ☐ 1 ☐ 2 ☐ 3 ☐ 4 ☐ 5

**42. Doctor's / dietitian's recommendation:** ☐ 1 ☐ 2 ☐ 3 ☐ 4 ☐ 5

**43. Affordable price:** ☐ 1 ☐ 2 ☐ 3 ☐ 4 ☐ 5

**Section 11: concerns and barriers**

**Rate your level of concern:**

**Scale:** 1 = Not concerned | 5 = Extremely concerned

**44. Safety and potential side effects:** ☐ 1 ☐ 2 ☐ 3 ☐ 4 ☐ 5

**45. Lack of long-term research evidence:** ☐ 1 ☐ 2 ☐ 3 ☐ 4 ☐ 5

**46. 46.** Higher cost:

*Note: recorded as binary response due to survey platform formatting.*

**47. Uncertainty about whether it will work:** ☐ 1 ☐ 2 ☐ 3 ☐ 4 ☐ 5

**48. Possible contamination (heavy metals):** ☐ 1 ☐ 2 ☐ 3 ☐ 4 ☐ 5

**Section 12: your beliefs about anemia treatment**

**Rate your agreement:** 1 = Strongly disagree | 5 = Strongly agree

**49.** My anemia seriously affects my quality of life.

☐ 1 ☐ 2 ☐ 3 ☐ 4 ☐ 5

**50.** Untreated anemia could lead to serious health problems.

☐ 1 ☐ 2 ☐ 3 ☐ 4 ☐ 5

**51.** Microalgae supplements could reduce my side effects.

☐ 1 ☐ 2 ☐ 3 ☐ 4 ☐ 5

**52.** I am confident I could use microalgae supplements correctly.

☐ 1 ☐ 2 ☐ 3 ☐ 4 ☐ 5

**53.** I could discuss this option with my doctor/dietitian.

☐ 1 ☐ 2 ☐ 3 ☐ 4 ☐ 5

**Section 13: cost, purchase & trust**

**54.** Maximum you would pay per month for microalgae supplements:

Less than 50 lei

☐ 50–99 lei

☐ 100–149 lei

☐ 150–199 lei

☐ 200–299 lei

☐ 300 lei or more

**55.** If proven effective but costing 2–3× more than current supplements, would you switch?

Definitely yes

☐ Probably yes

☐ Unsure

☐ Probably no

☐ Definitely no

**56.** Preferred form:

Tablets/pills

☐ Capsules

☐ Powder (mix in drinks)

☐ Liquid

☐ No preference

**57.** Where would you prefer to buy it?

Pharmacy

☐ Online

☐ Health food store

☐ Doctor's office/dietitian's practice

☐ Supermarket

**58.** I trust my doctor's recommendations regarding supplements.

**Scale:** 1 = Strongly disagree | 5 = Strongly agree

☐ 1 ☐ 2 ☐ 3 ☐ 4 ☐ 5

**59.** I trust scientific research as a basis for health decisions.

**Scale:** 1 = Strongly disagree | 5 = Strongly agree

☐ 1 ☐ 2 ☐ 3 ☐ 4 ☐ 5

**60.** What level of scientific evidence would you need before trying microalgae supplements?

None — willing to try based on this description

☐ At least one published study

☐ Multiple published studies

☐ Large-scale clinical trials

☐ Regulatory approval (e.g., EFSA/EU)

☐ 5+ years of safety data

**Section 14: open questions**

**61.** What is your biggest concern about trying microalgae-based iron supplements?

Your answer: _______________________________________

**62.** What would make you more likely to try them?

Your answer: _______________________________________

**63.** Any other comments?

Your answer: _______________________________________

**Thank you!**

Your responses will help researchers develop better-tolerated iron supplementation options.

If you have any questions about this research, please contact: **alexandra.lacurezeanu@student.usamvcluj.ro**
